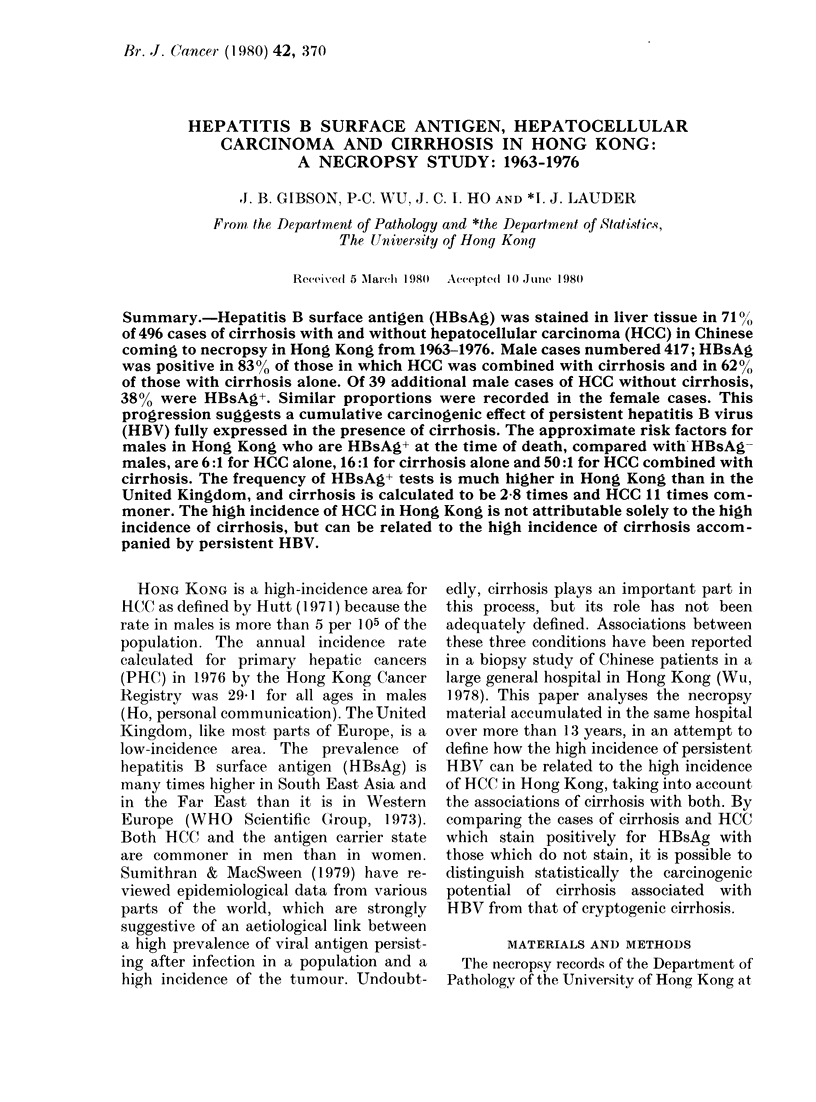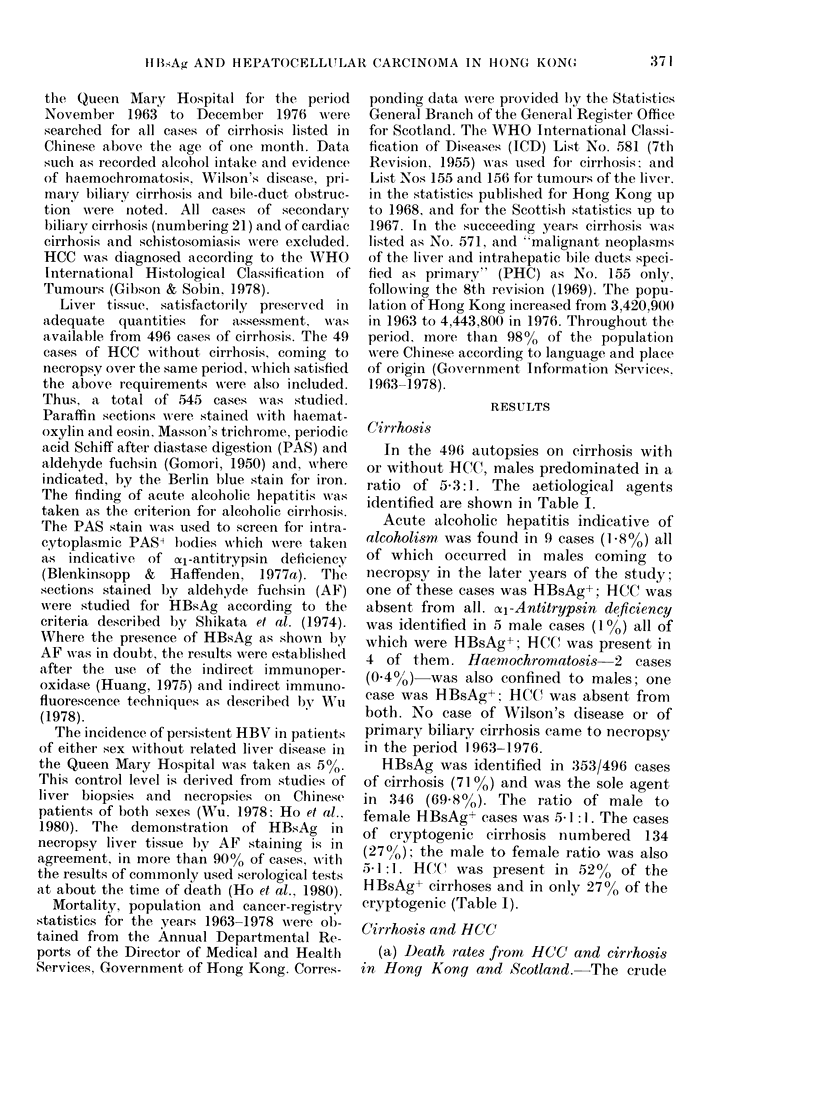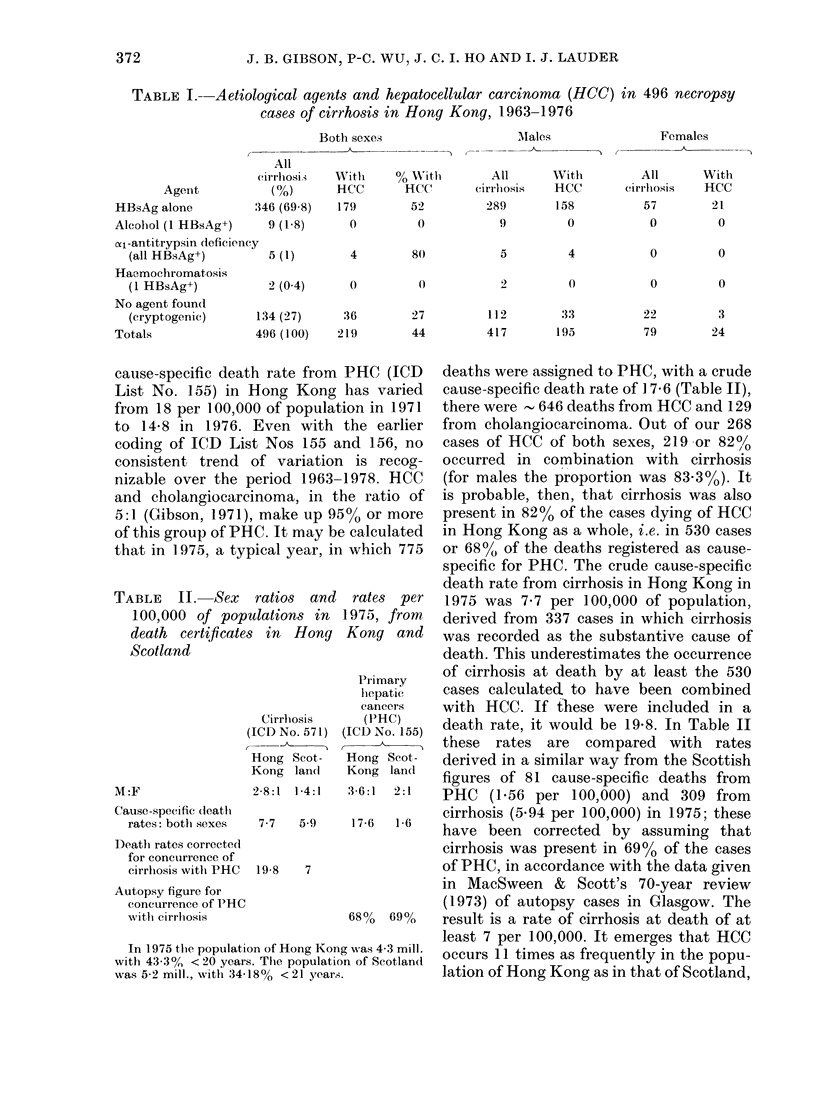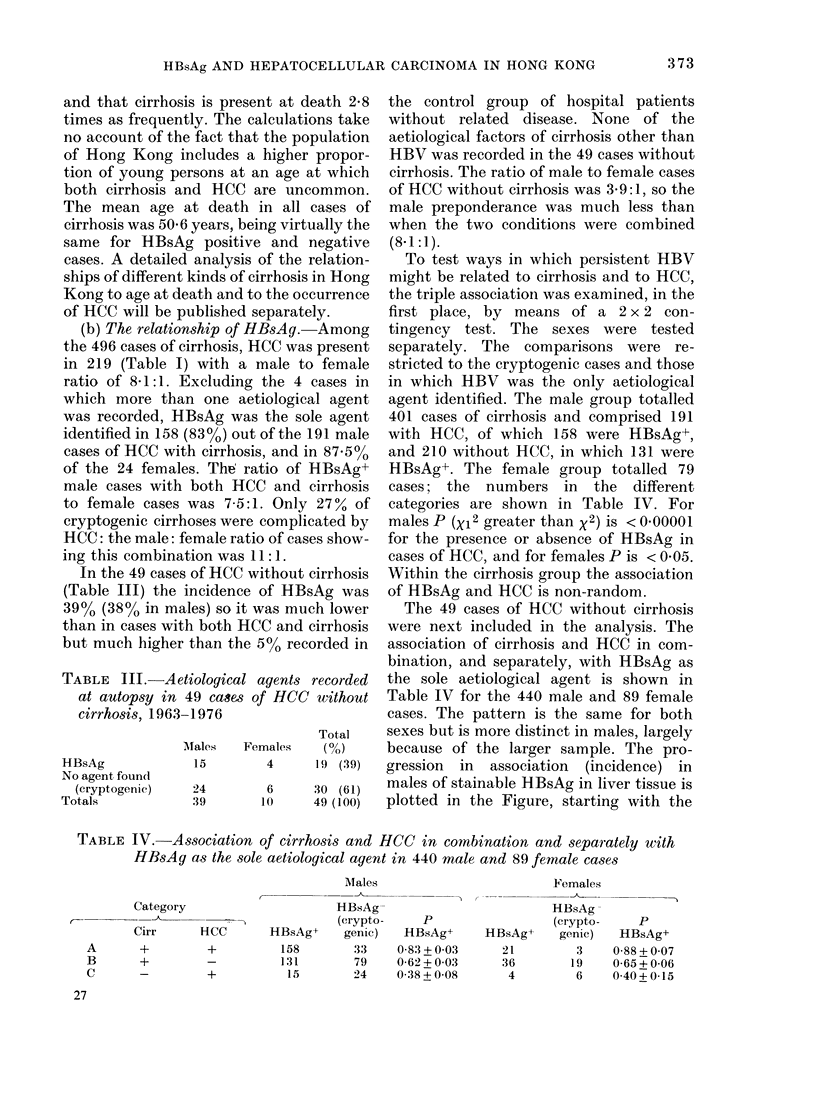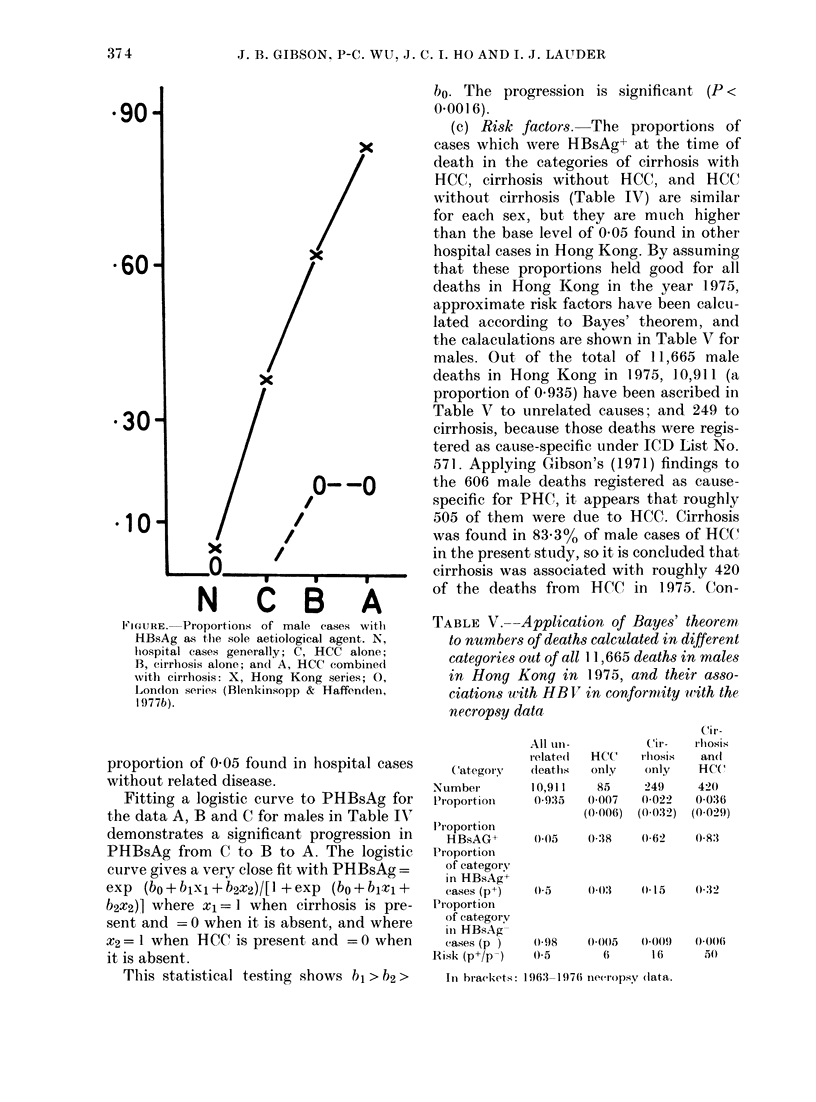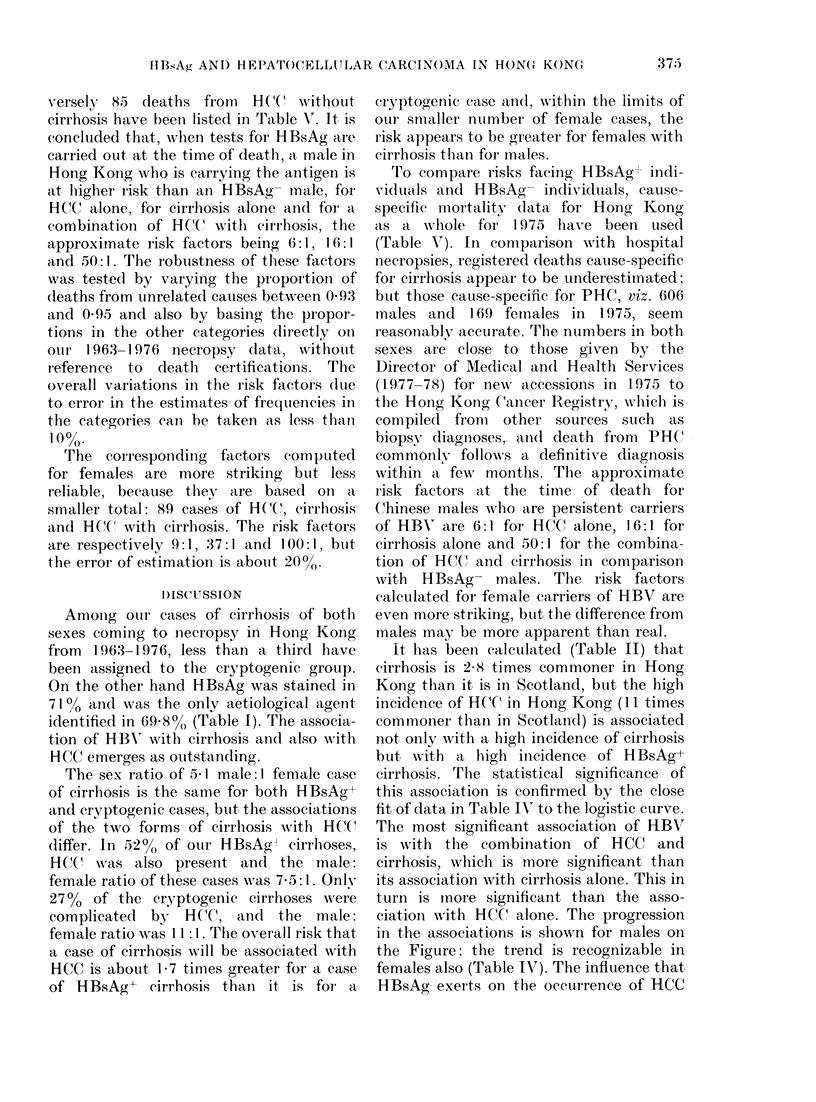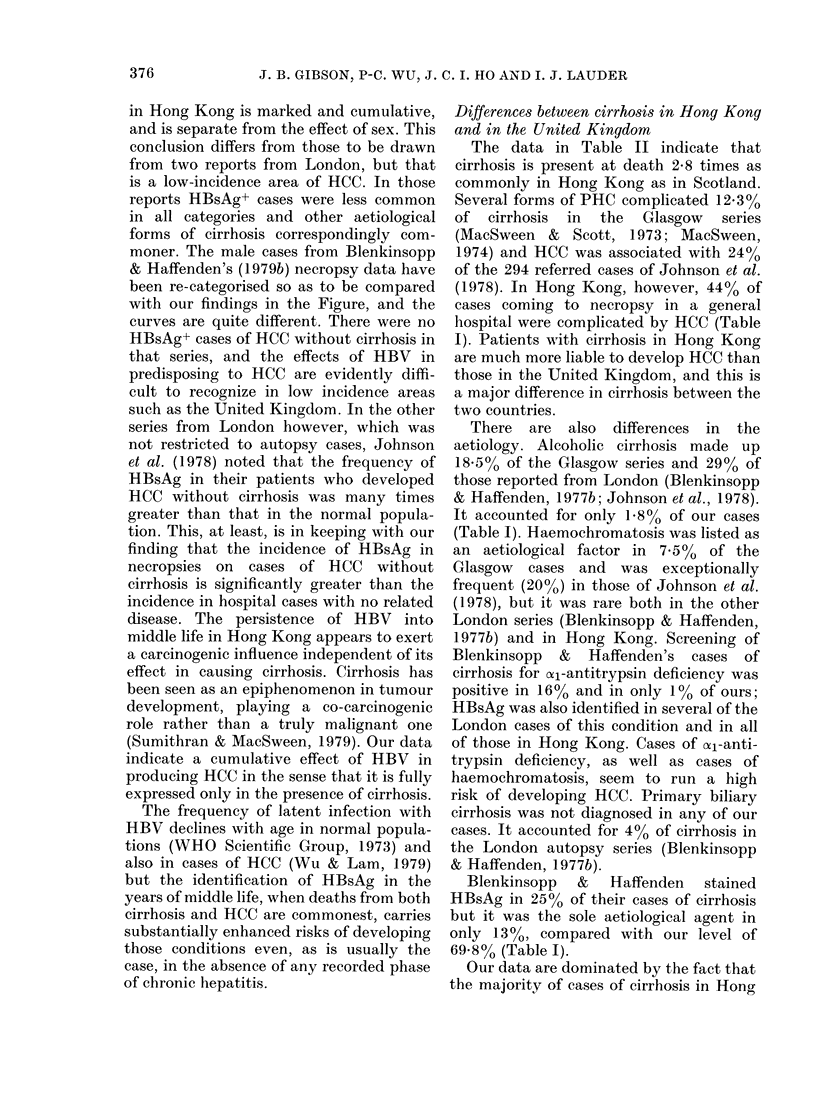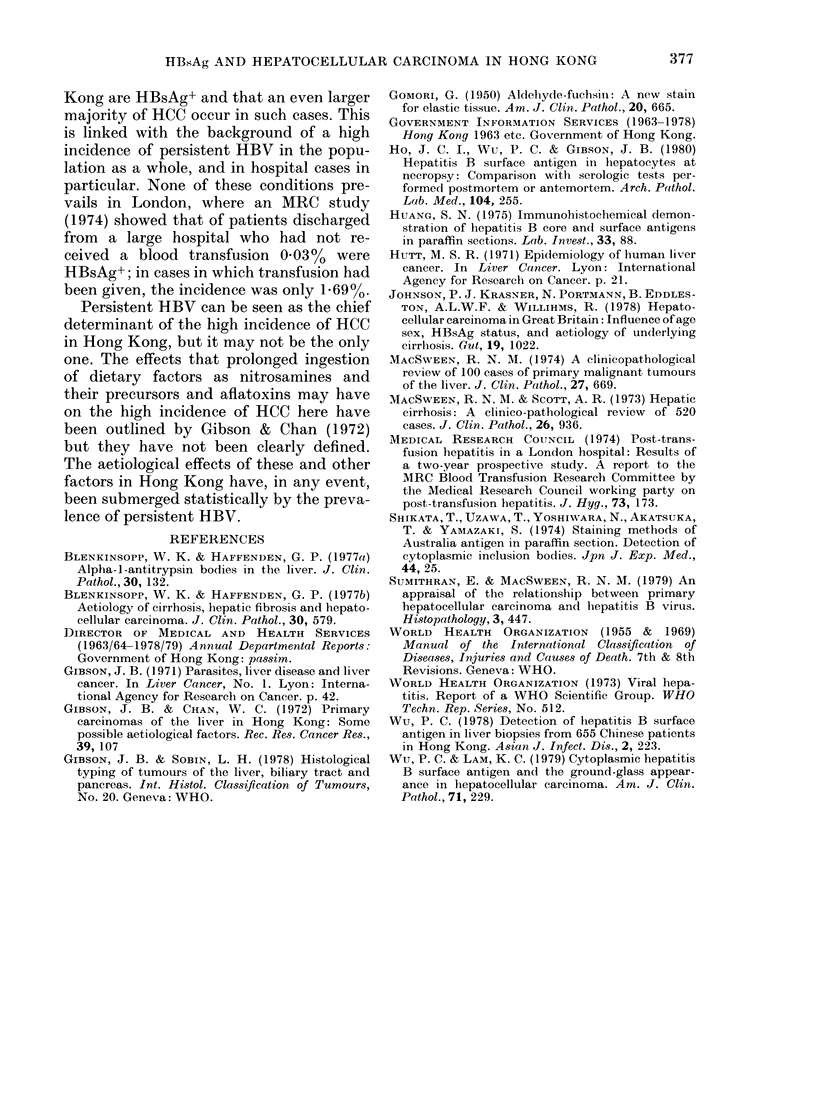# Hepatitis B surface antigen, hepatocellular carcinoma and cirrhosis in hong kong: a necropsy study: 1963-1976.

**DOI:** 10.1038/bjc.1980.247

**Published:** 1980-09

**Authors:** J. B. Gibson, P. C. Wu, J. C. Ho, I. J. Lauder

## Abstract

Hepatitis B surface antigen (HBsAg) was stained in liver tissue in 71% of 496 cases of cirrhosis with and without hepatocellular carcinoma (HCC) in Chinese coming to necropsy in Hong Kong from 1963-1976. Male cases numbered 417; HBsAg was positive in 83% of those in which HCC was combined with cirrhosis and in 62% of those with cirrhosis alone. Of 39 additional male cases of HCC without cirrhosis, 38% were HBsAg+. Similar proportions were recorded in the famale cases. This progression suggests a cumulative carcinogenic effect of persistent hepatitis B virus (HBV) fully expressed in the presence of cirrhosis. The approximate risk factors for males in Hong Kong who are HBsAg+ at the time of death, compared with HBsAg- males, are 6:1 for HCC alone, 16:1 for cirrhosis alone and 50:1 for HCC combined with cirrhosis. The frequency of HBsAg+ tests is much higher in Hong Kong than in the United Kingdom, and cirrhosis is calculated to be 2.8 times and HCC 11 times commoner. The high incidence of HCC in Hong Kong is not attributable solely to the high incidence of cirrhosis, but can be related to the high incidence of cirrhosis accompanied by persistent HBV.


Br. J. Cancer (1980) 42, 370

### HEPATITIS B SURFACE ANTIGEN, HEPATOCELLULAR CARCINOMA AND CIRRHOSIS IN HONG KONG: A NECROPSY STUDY: 1963-1976

J. B. GIBSON, P-C. WU, J. C. I. HO AND *I. J. LAUDER

*From the Department of Pathology and *the Department of Statistics, The University of Hong Kong*

Received 5 March 1980 Accepted 10 June 1980

**Summary.—Hepatitis B surface antigen (HBsAg) was stained in liver tissue in 71% of 496 cases of cirrhosis with and without hepatocellular carcinoma (HCC) in Chinese coming to necropsy in Hong Kong from 1963–1976. Male cases numbered 417; HBsAg was positive in 83% of those in which HCC was combined with cirrhosis and in 62% of those with cirrhosis alone. Of 39 additional male cases of HCC without cirrhosis, 38% were HBsAg$^+$. Similar proportions were recorded in the female cases. This progression suggests a cumulative carcinogenic effect of persistent hepatitis B virus (HBV) fully expressed in the presence of cirrhosis. The approximate risk factors for males in Hong Kong who are HBsAg$^+$ at the time of death, compared with HBsAg$^-$ males, are 6:1 for HCC alone, 16:1 for cirrhosis alone and 50:1 for HCC combined with cirrhosis. The frequency of HBsAg$^+$ tests is much higher in Hong Kong than in the United Kingdom, and cirrhosis is calculated to be 2·8 times and HCC 11 times commoner. The high incidence of HCC in Hong Kong is not attributable solely to the high incidence of cirrhosis, but can be related to the high incidence of cirrhosis accompanied by persistent HBV.**

HONG KONG is a high-incidence area for HCC as defined by Hutt (1971) because the rate in males is more than 5 per $10^5$ of the population. The annual incidence rate calculated for primary hepatic cancers (PHC) in 1976 by the Hong Kong Cancer Registry was 29·1 for all ages in males (Ho, personal communication). The United Kingdom, like most parts of Europe, is a low-incidence area. The prevalence of hepatitis B surface antigen (HBsAg) is many times higher in South East Asia and in the Far East than it is in Western Europe (WHO Scientific Group, 1973). Both HCC and the antigen carrier state are commoner in men than in women. Sumithran & MacSween (1979) have reviewed epidemiological data from various parts of the world, which are strongly suggestive of an aetiological link between a high prevalence of viral antigen persisting after infection in a population and a high incidence of the tumour. Undoubtedly, cirrhosis plays an important part in this process, but its role has not been adequately defined. Associations between these three conditions have been reported in a biopsy study of Chinese patients in a large general hospital in Hong Kong (Wu, 1978). This paper analyses the necropsy material accumulated in the same hospital over more than 13 years, in an attempt to define how the high incidence of persistent HBV can be related to the high incidence of HCC in Hong Kong, taking into account the associations of cirrhosis with both. By comparing the cases of cirrhosis and HCC which stain positively for HBsAg with those which do not stain, it is possible to distinguish statistically the carcinogenic potential of cirrhosis associated with HBV from that of cryptogenic cirrhosis.

### MATERIALS AND METHODS

The necropsy records of the Department of Pathology of the University of Hong Kong at the Queen Mary Hospital for the period November 1963 to December 1976 were searched for all cases of cirrhosis listed in Chinese above the age of one month. Data such as recorded alcohol intake and evidence of haemochromatosis, Wilson's disease, primary biliary cirrhosis and bile-duct obstruction were noted. All cases of secondary biliary cirrhosis (numbering 21) and of cardiac cirrhosis and schistosomiasis were excluded. HCC was diagnosed according to the WHO International Histological Classification of Tumours (Gibson & Sobin, 1978).

Liver tissue, satisfactorily preserved in adequate quantities for assessment, was available from 496 cases of cirrhosis. The 49 cases of HCC without cirrhosis, coming to necropsy over the same period, which satisfied the above requirements were also included. Thus, a total of 545 cases was studied. Paraffin sections were stained with haematoxylin and eosin, Masson's trichrome, periodic acid Schiff after diastase digestion (PAS) and aldehyde fuchsin (Gomori, 1950) and, where indicated, by the Berlin blue stain for iron. The finding of acute alcoholic hepatitis was taken as the criterion for alcoholic cirrhosis. The PAS stain was used to screen for intracytoplasmic $PAS^+$ bodies which were taken as indicative of $\alpha_1$-antitrypsin deficiency (Blenkinsopp & Haffenden, 1977*a*). The sections stained by aldehyde fuchsin (AF) were studied for HBsAg according to the criteria described by Shikata *et al.* (1974). Where the presence of HBsAg as shown by AF was in doubt, the results were established after the use of the indirect immunoperoxidase (Huang, 1975) and indirect immunofluorescence techniques as described by Wu (1978).

The incidence of persistent HBV in patients of either sex without related liver disease in the Queen Mary Hospital was taken as 5%. This control level is derived from studies of liver biopsies and necropsies on Chinese patients of both sexes (Wu, 1978; Ho *et al.*, 1980). The demonstration of HBsAg in necropsy liver tissue by AF staining is in agreement, in more than 90% of cases, with the results of commonly used serological tests at about the time of death (Ho *et al.*, 1980).

Mortality, population and cancer-registry statistics for the years 1963–1978 were obtained from the Annual Departmental Reports of the Director of Medical and Health Services, Government of Hong Kong. Corresponding data were provided by the Statistics General Branch of the General Register Office for Scotland. The WHO International Classification of Diseases (ICD) List No. 581 (7th Revision, 1955) was used for cirrhosis; and List Nos 155 and 156 for tumours of the liver, in the statistics published for Hong Kong up to 1968, and for the Scottish statistics up to 1967. In the succeeding years cirrhosis was listed as No. 571, and "malignant neoplasms of the liver and intrahepatic bile ducts specified as primary" (PHC) as No. 155 only, following the 8th revision (1969). The population of Hong Kong increased from 3,420,900 in 1963 to 4,443,800 in 1976. Throughout the period, more than 98% of the population were Chinese according to language and place of origin (Government Information Services, 1963–1978).

### RESULTS

### *Cirrhosis*

In the 496 autopsies on cirrhosis with or without HCC, males predominated in a ratio of 5·3:1. The aetiological agents identified are shown in Table I.

Acute alcoholic hepatitis indicative of *alcoholism* was found in 9 cases (1·8%) all of which occurred in males coming to necropsy in the later years of the study; one of these cases was $HBsAg^+$; HCC was absent from all. $\alpha_1$*-Antitrypsin deficiency* was identified in 5 male cases (1%) all of which were $HBsAg^+$; HCC was present in 4 of them. *Haemochromatosis*—2 cases (0·4%)—was also confined to males; one case was $HBsAg^+$; HCC was absent from both. No case of Wilson's disease or of primary biliary cirrhosis came to necropsy in the period 1963–1976.

HBsAg was identified in 353/496 cases of cirrhosis (71%) and was the sole agent in 346 (69·8%). The ratio of male to female $HBsAg^+$ cases was 5·1:1. The cases of cryptogenic cirrhosis numbered 134 (27%); the male to female ratio was also 5·1:1. HCC was present in 52% of the $HBsAg^+$ cirrhoses and in only 27% of the cryptogenic (Table I).

### *Cirrhosis and HCC*

(a) *Death rates from HCC and cirrhosis in Hong Kong and Scotland.*—The crude

TABLE I.—*Aetiological agents and hepatocellular carcinoma (HCC) in 496 necropsy cases of cirrhosis in Hong Kong*, 1963–1976

| | Both sexes | | | Males | | Females | |
|---|---|---|---|---|---|---|---|
| Agent | All cirrhosis (%) | With HCC | % With HCC | All cirrhosis | With HCC | All cirrhosis | With HCC |
| HBsAg alone | 346 (69·8) | 179 | 52 | 289 | 158 | 57 | 21 |
| Alcohol (1 HBsAg+) | 9 (1·8) | 0 | 0 | 9 | 0 | 0 | 0 |
| $\alpha_1$-antitrypsin deficiency (all HBsAg+) | 5 (1) | 4 | 80 | 5 | 4 | 0 | 0 |
| Haemochromatosis (1 HBsAg+) | 2 (0·4) | 0 | 0 | 2 | 0 | 0 | 0 |
| No agent found (cryptogenic) | 134 (27) | 36 | 27 | 112 | 33 | 22 | 3 |
| Totals | 496 (100) | 219 | 44 | 417 | 195 | 79 | 24 |

cause-specific death rate from PHC (ICD List No. 155) in Hong Kong has varied from 18 per 100,000 of population in 1971 to 14·8 in 1976. Even with the earlier coding of ICD List Nos 155 and 156, no consistent trend of variation is recognizable over the period 1963–1978. HCC and cholangiocarcinoma, in the ratio of 5:1 (Gibson, 1971), make up 95% or more of this group of PHC. It may be calculated that in 1975, a typical year, in which 775 deaths were assigned to PHC, with a crude cause-specific death rate of 17·6 (Table II), there were ~ 646 deaths from HCC and 129 from cholangiocarcinoma. Out of our 268 cases of HCC of both sexes, 219 or 82% occurred in combination with cirrhosis (for males the proportion was 83·3%). It is probable, then, that cirrhosis was also present in 82% of the cases dying of HCC in Hong Kong as a whole, *i.e.* in 530 cases or 68% of the deaths registered as cause-specific for PHC. The crude cause-specific death rate from cirrhosis in Hong Kong in 1975 was 7·7 per 100,000 of population, derived from 337 cases in which cirrhosis was recorded as the substantive cause of death. This underestimates the occurrence of cirrhosis at death by at least the 530 cases calculated to have been combined with HCC. If these were included in a death rate, it would be 19·8. In Table II these rates are compared with rates derived in a similar way from the Scottish figures of 81 cause-specific deaths from PHC (1·56 per 100,000) and 309 from cirrhosis (5·94 per 100,000) in 1975; these have been corrected by assuming that cirrhosis was present in 69% of the cases of PHC, in accordance with the data given in MacSween & Scott's 70-year review (1973) of autopsy cases in Glasgow. The result is a rate of cirrhosis at death of at least 7 per 100,000. It emerges that HCC occurs 11 times as frequently in the population of Hong Kong as in that of Scotland,

TABLE II.—*Sex ratios and rates per* 100,000 *of populations in* 1975, *from death certificates in Hong Kong and Scotland*

| | Cirrhosis (ICD No. 571) | | Primary hepatic cancers (PHC) (ICD No. 155) | |
|---|---|---|---|---|
| | Hong Kong | Scotland | Hong Kong | Scotland |
| M:F | 2·8:1 | 1·4:1 | 3·6:1 | 2:1 |
| Cause-specific death rates: both sexes | 7·7 | 5·9 | 17·6 | 1·6 |
| Death rates corrected for concurrence of cirrhosis with PHC | 19·8 | 7 | | |
| Autopsy figure for concurrence of PHC with cirrhosis | | | 68% | 69% |

In 1975 the population of Hong Kong was 4·3 mill. with 43·3% < 20 years. The population of Scotland was 5·2 mill., with 34·18% < 21 years.

and that cirrhosis is present at death 2·8 times as frequently. The calculations take no account of the fact that the population of Hong Kong includes a higher proportion of young persons at an age at which both cirrhosis and HCC are uncommon. The mean age at death in all cases of cirrhosis was 50·6 years, being virtually the same for HBsAg positive and negative cases. A detailed analysis of the relationships of different kinds of cirrhosis in Hong Kong to age at death and to the occurrence of HCC will be published separately.

(b) *The relationship of HBsAg.*—Among the 496 cases of cirrhosis, HCC was present in 219 (Table I) with a male to female ratio of 8·1:1. Excluding the 4 cases in which more than one aetiological agent was recorded, HBsAg was the sole agent identified in 158 (83%) out of the 191 male cases of HCC with cirrhosis, and in 87·5% of the 24 females. The ratio of $HBsAg^+$ male cases with both HCC and cirrhosis to female cases was 7·5:1. Only 27% of cryptogenic cirrhoses were complicated by HCC: the male: female ratio of cases showing this combination was 11:1.

In the 49 cases of HCC without cirrhosis (Table III) the incidence of HBsAg was 39% (38% in males) so it was much lower than in cases with both HCC and cirrhosis but much higher than the 5% recorded in the control group of hospital patients without related disease. None of the aetiological factors of cirrhosis other than HBV was recorded in the 49 cases without cirrhosis. The ratio of male to female cases of HCC without cirrhosis was 3·9:1, so the male preponderance was much less than when the two conditions were combined (8·1:1).

TABLE III.—*Aetiological agents recorded at autopsy in* 49 *cases of HCC without cirrhosis*, 1963–1976

| | Males | Females | Total (%) |
|---|---|---|---|
| HBsAg | 15 | 4 | 19 (39) |
| No agent found (cryptogenic) | 24 | 6 | 30 (61) |
| Totals | 39 | 10 | 49 (100) |

To test ways in which persistent HBV might be related to cirrhosis and to HCC, the triple association was examined, in the first place, by means of a 2 × 2 contingency test. The sexes were tested separately. The comparisons were restricted to the cryptogenic cases and those in which HBV was the only aetiological agent identified. The male group totalled 401 cases of cirrhosis and comprised 191 with HCC, of which 158 were $HBsAg^+$, and 210 without HCC, in which 131 were $HBsAg^+$. The female group totalled 79 cases; the numbers in the different categories are shown in Table IV. For males $P$ (${\chi_1}^2$ greater than $\chi^2$) is $< 0{\cdot}00001$ for the presence or absence of HBsAg in cases of HCC, and for females $P$ is $< 0{\cdot}05$. Within the cirrhosis group the association of HBsAg and HCC is non-random.

The 49 cases of HCC without cirrhosis were next included in the analysis. The association of cirrhosis and HCC in combination, and separately, with HBsAg as the sole aetiological agent is shown in Table IV for the 440 male and 89 female cases. The pattern is the same for both sexes but is more distinct in males, largely because of the larger sample. The progression in association (incidence) in males of stainable HBsAg in liver tissue is plotted in the Figure, starting with the

TABLE IV.—*Association of cirrhosis and HCC in combination and separately with HBsAg as the sole aetiological agent in* 440 *male and* 89 *female cases*

| | Category | | Males | | | Females | | |
|---|---|---|---|---|---|---|---|---|
| | Cirr | HCC | $HBsAg^+$ | $HBsAg^-$ (cryptogenic) | $P$ $HBsAg^+$ | $HBsAg^+$ | $HBsAg^-$ (cryptogenic) | $P$ $HBsAg^+$ |
| A | + | + | 158 | 33 | $0{\cdot}83 \pm 0{\cdot}03$ | 21 | 3 | $0{\cdot}88 \pm 0{\cdot}07$ |
| B | + | − | 131 | 79 | $0{\cdot}62 \pm 0{\cdot}03$ | 36 | 19 | $0{\cdot}65 \pm 0{\cdot}06$ |
| C | − | + | 15 | 24 | $0{\cdot}38 \pm 0{\cdot}08$ | 4 | 6 | $0{\cdot}40 \pm 0{\cdot}15$ |

FIGURE.—Proportions of male cases with HBsAg as the sole aetiological agent. N, hospital cases generally; C, HCC alone; B, cirrhosis alone; and A, HCC combined with cirrhosis: X, Hong Kong series; O, London series (Blenkinsopp & Haffenden, 1977*b*).

proportion of 0·05 found in hospital cases without related disease.

Fitting a logistic curve to PHBsAg for the data A, B and C for males in Table IV demonstrates a significant progression in PHBsAg from C to B to A. The logistic curve gives a very close fit with PHBsAg = exp $(b_0+b_1x_1+b_2x_2)/[1+\exp(b_0+b_1x_1+b_2x_2)]$ where $x_1=1$ when cirrhosis is present and $=0$ when it is absent, and where $x_2=1$ when HCC is present and $=0$ when it is absent.

This statistical testing shows $b_1>b_2>b_0$. The progression is significant ($P<0{\cdot}0016$).

(c) *Risk factors.*—The proportions of cases which were HBsAg$^+$ at the time of death in the categories of cirrhosis with HCC, cirrhosis without HCC, and HCC without cirrhosis (Table IV) are similar for each sex, but they are much higher than the base level of 0·05 found in other hospital cases in Hong Kong. By assuming that these proportions held good for all deaths in Hong Kong in the year 1975, approximate risk factors have been calculated according to Bayes' theorem, and the calaculations are shown in Table V for males. Out of the total of 11,665 male deaths in Hong Kong in 1975, 10,911 (a proportion of 0·935) have been ascribed in Table V to unrelated causes; and 249 to cirrhosis, because those deaths were registered as cause-specific under ICD List No. 571. Applying Gibson's (1971) findings to the 606 male deaths registered as cause-specific for PHC, it appears that roughly 505 of them were due to HCC. Cirrhosis was found in 83·3% of male cases of HCC in the present study, so it is concluded that cirrhosis was associated with roughly 420 of the deaths from HCC in 1975. Con-

TABLE V.—*Application of Bayes' theorem to numbers of deaths calculated in different categories out of all* 11,665 *deaths in males in Hong Kong in* 1975, *and their associations with HBV in conformity with the necropsy data*

| Category | All unrelated deaths | HCC only | Cirrhosis only | Cirrhosis and HCC |
|---|---|---|---|---|
| Number | 10,911 | 85 | 249 | 420 |
| Proportion | 0·935 | 0·007 (0·006) | 0·022 (0·032) | 0·036 (0·029) |
| Proportion HBsAG$^+$ | 0·05 | 0·38 | 0·62 | 0·83 |
| Proportion of category in HBsAg$^+$ cases ($p^+$) | 0·5 | 0·03 | 0·15 | 0·32 |
| Proportion of category in HBsAg$^-$ cases ($p^-$) | 0·98 | 0·005 | 0·009 | 0·006 |
| Risk ($p^+/p^-$) | 0·5 | 6 | 16 | 50 |

In brackets: 1963–1976 necropsy data.

versely 85 deaths from HCC without cirrhosis have been listed in Table V. It is concluded that, when tests for HBsAg are carried out at the time of death, a male in Hong Kong who is carrying the antigen is at higher risk than an $HBsAg^-$ male, for HCC alone, for cirrhosis alone and for a combination of HCC with cirrhosis, the approximate risk factors being 6:1, 16:1 and 50:1. The robustness of these factors was tested by varying the proportion of deaths from unrelated causes between 0·93 and 0·95 and also by basing the proportions in the other categories directly on our 1963–1976 necropsy data, without reference to death certifications. The overall variations in the risk factors due to error in the estimates of frequencies in the categories can be taken as less than 10%.

The corresponding factors computed for females are more striking but less reliable, because they are based on a smaller total: 89 cases of HCC, cirrhosis and HCC with cirrhosis. The risk factors are respectively 9:1, 37:1 and 100:1, but the error of estimation is about 20%.

### DISCUSSION

Among our cases of cirrhosis of both sexes coming to necropsy in Hong Kong from 1963–1976, less than a third have been assigned to the cryptogenic group. On the other hand HBsAg was stained in 71% and was the only aetiological agent identified in 69·8% (Table I). The association of HBV with cirrhosis and also with HCC emerges as outstanding.

The sex ratio of 5·1 male:1 female case of cirrhosis is the same for both $HBsAg^+$ and cryptogenic cases, but the associations of the two forms of cirrhosis with HCC differ. In 52% of our $HBsAg^+$ cirrhoses, HCC was also present and the male: female ratio of these cases was 7·5:1. Only 27% of the cryptogenic cirrhoses were complicated by HCC, and the male: female ratio was 11:1. The overall risk that a case of cirrhosis will be associated with HCC is about 1·7 times greater for a case of $HBsAg^+$ cirrhosis than it is for a cryptogenic case and, within the limits of our smaller number of female cases, the risk appears to be greater for females with cirrhosis than for males.

To compare risks facing $HBsAg^+$ individuals and $HBsAg^-$ individuals, cause-specific mortality data for Hong Kong as a whole for 1975 have been used (Table V). In comparison with hospital necropsies, registered deaths cause-specific for cirrhosis appear to be underestimated; but those cause-specific for PHC, *viz.* 606 males and 169 females in 1975, seem reasonably accurate. The numbers in both sexes are close to those given by the Director of Medical and Health Services (1977–78) for new accessions in 1975 to the Hong Kong Cancer Registry, which is compiled from other sources such as biopsy diagnoses, and death from PHC commonly follows a definitive diagnosis within a few months. The approximate risk factors at the time of death for Chinese males who are persistent carriers of HBV are 6:1 for HCC alone, 16:1 for cirrhosis alone and 50:1 for the combination of HCC and cirrhosis in comparison with $HBsAg^-$ males. The risk factors calculated for female carriers of HBV are even more striking, but the difference from males may be more apparent than real.

It has been calculated (Table II) that cirrhosis is 2·8 times commoner in Hong Kong than it is in Scotland, but the high incidence of HCC in Hong Kong (11 times commoner than in Scotland) is associated not only with a high incidence of cirrhosis but with a high incidence of $HBsAg^+$ cirrhosis. The statistical significance of this association is confirmed by the close fit of data in Table IV to the logistic curve. The most significant association of HBV is with the combination of HCC and cirrhosis, which is more significant than its association with cirrhosis alone. This in turn is more significant than the association with HCC alone. The progression in the associations is shown for males on the Figure; the trend is recognizable in females also (Table IV). The influence that HBsAg exerts on the occurrence of HCC

in Hong Kong is marked and cumulative, and is separate from the effect of sex. This conclusion differs from those to be drawn from two reports from London, but that is a low-incidence area of HCC. In those reports HBsAg$^+$ cases were less common in all categories and other aetiological forms of cirrhosis correspondingly commoner. The male cases from Blenkinsopp & Haffenden's (1979*b*) necropsy data have been re-categorised so as to be compared with our findings in the Figure, and the curves are quite different. There were no HBsAg$^+$ cases of HCC without cirrhosis in that series, and the effects of HBV in predisposing to HCC are evidently difficult to recognize in low incidence areas such as the United Kingdom. In the other series from London however, which was not restricted to autopsy cases, Johnson *et al.* (1978) noted that the frequency of HBsAg in their patients who developed HCC without cirrhosis was many times greater than that in the normal population. This, at least, is in keeping with our finding that the incidence of HBsAg in necropsies on cases of HCC without cirrhosis is significantly greater than the incidence in hospital cases with no related disease. The persistence of HBV into middle life in Hong Kong appears to exert a carcinogenic influence independent of its effect in causing cirrhosis. Cirrhosis has been seen as an epiphenomenon in tumour development, playing a co-carcinogenic role rather than a truly malignant one (Sumithran & MacSween, 1979). Our data indicate a cumulative effect of HBV in producing HCC in the sense that it is fully expressed only in the presence of cirrhosis.

The frequency of latent infection with HBV declines with age in normal populations (WHO Scientific Group, 1973) and also in cases of HCC (Wu & Lam, 1979) but the identification of HBsAg in the years of middle life, when deaths from both cirrhosis and HCC are commonest, carries substantially enhanced risks of developing those conditions even, as is usually the case, in the absence of any recorded phase of chronic hepatitis.

*Differences between cirrhosis in Hong Kong and in the United Kingdom*

The data in Table II indicate that cirrhosis is present at death 2·8 times as commonly in Hong Kong as in Scotland. Several forms of PHC complicated 12·3% of cirrhosis in the Glasgow series (MacSween & Scott, 1973; MacSween, 1974) and HCC was associated with 24% of the 294 referred cases of Johnson *et al.* (1978). In Hong Kong, however, 44% of cases coming to necropsy in a general hospital were complicated by HCC (Table I). Patients with cirrhosis in Hong Kong are much more liable to develop HCC than those in the United Kingdom, and this is a major difference in cirrhosis between the two countries.

There are also differences in the aetiology. Alcoholic cirrhosis made up 18·5% of the Glasgow series and 29% of those reported from London (Blenkinsopp & Haffenden, 1977*b*; Johnson *et al.*, 1978). It accounted for only 1·8% of our cases (Table I). Haemochromatosis was listed as an aetiological factor in 7·5% of the Glasgow cases and was exceptionally frequent (20%) in those of Johnson *et al.* (1978), but it was rare both in the other London series (Blenkinsopp & Haffenden, 1977*b*) and in Hong Kong. Screening of Blenkinsopp & Haffenden's cases of cirrhosis for $\alpha_1$-antitrypsin deficiency was positive in 16% and in only 1% of ours; HBsAg was also identified in several of the London cases of this condition and in all of those in Hong Kong. Cases of $\alpha_1$-antitrypsin deficiency, as well as cases of haemochromatosis, seem to run a high risk of developing HCC. Primary biliary cirrhosis was not diagnosed in any of our cases. It accounted for 4% of cirrhosis in the London autopsy series (Blenkinsopp & Haffenden, 1977*b*).

Blenkinsopp & Haffenden stained HBsAg in 25% of their cases of cirrhosis but it was the sole aetiological agent in only 13%, compared with our level of 69·8% (Table I).

Our data are dominated by the fact that the majority of cases of cirrhosis in Hong

Kong are $HBsAg^+$ and that an even larger majority of HCC occur in such cases. This is linked with the background of a high incidence of persistent HBV in the population as a whole, and in hospital cases in particular. None of these conditions prevails in London, where an MRC study (1974) showed that of patients discharged from a large hospital who had not received a blood transfusion 0·03% were $HBsAg^+$; in cases in which transfusion had been given, the incidence was only 1·69%.

Persistent HBV can be seen as the chief determinant of the high incidence of HCC in Hong Kong, but it may not be the only one. The effects that prolonged ingestion of dietary factors as nitrosamines and their precursors and aflatoxins may have on the high incidence of HCC here have been outlined by Gibson & Chan (1972) but they have not been clearly defined. The aetiological effects of these and other factors in Hong Kong have, in any event, been submerged statistically by the prevalence of persistent HBV.

### REFERENCES

BLENKINSOPP, W. K. & HAFFENDEN, G. P. (1977*a*) Alpha-1-antitrypsin bodies in the liver. *J. Clin. Pathol.*, **30**, 132.

BLENKINSOPP, W. K. & HAFFENDEN, G. P. (1977*b*) Aetiology of cirrhosis, hepatic fibrosis and hepatocellular carcinoma. *J. Clin. Pathol.*, **30**, 579.

DIRECTOR OF MEDICAL AND HEALTH SERVICES (1963/64–1978/79) *Annual Departmental Reports:* Government of Hong Kong: *passim.*

GIBSON, J. B. (1971) Parasites, liver disease and liver cancer. In *Liver Cancer*, No. 1. Lyon: International Agency for Research on Cancer. p. 42.

GIBSON, J. B. & CHAN, W. C. (1972) Primary carcinomas of the liver in Hong Kong: Some possible aetiological factors. *Rec. Res. Cancer Res.*, **39**, 107

GIBSON, J. B. & SOBIN, L. H. (1978) Histological typing of tumours of the liver, biliary tract and pancreas. *Int. Histol. Classification of Tumours*, No. 20. Geneva: WHO.

GOMORI, G. (1950) Aldehyde-fuchsin: A new stain for elastic tissue. *Am. J. Clin. Pathol.*, **20**, 665.

GOVERNMENT INFORMATION SERVICES (1963–1978) *Hong Kong* 1963 etc. Government of Hong Kong.

HO, J. C. I., WU, P. C. & GIBSON, J. B. (1980) Hepatitis B surface antigen in hepatocytes at necropsy: Comparison with serologic tests performed postmortem or antemortem. *Arch. Pathol. Lab. Med.*, **104**, 255.

HUANG, S. N. (1975) Immunohistochemical demonstration of hepatitis B core and surface antigens in paraffin sections. *Lab. Invest.*, **33**, 88.

HUTT, M. S. R. (1971) Epidemiology of human liver cancer. In *Liver Cancer*. Lyon: International Agency for Research on Cancer. p. 21.

JOHNSON, P. J. KRASNER, N. PORTMANN, B. EDDLESTON, A.L.W.F. & WILLIHMS, R. (1978) Hepatocellular carcinoma in Great Britain: Influence of age sex, HBsAg status, and aetiology of underlying cirrhosis. *Gut*, **19**, 1022.

MACSWEEN, R. N. M. (1974) A clinicopathological review of 100 cases of primary malignant tumours of the liver. *J. Clin. Pathol.*, **27**, 669.

MACSWEEN, R. N. M. & SCOTT, A. R. (1973) Hepatic cirrhosis: A clinico-pathological review of 520 cases. *J. Clin. Pathol.*, **26**, 936.

MEDICAL RESEARCH COUNCIL (1974) Post-transfusion hepatitis in a London hospital: Results of a two-year prospective study. A report to the MRC Blood Transfusion Research Committee by the Medical Research Council working party on post-transfusion hepatitis. *J. Hyg.*, **73**, 173.

SHIKATA, T., UZAWA, T., YOSHIWARA, N., AKATSUKA, T. & YAMAZAKI, S. (1974) Staining methods of Australia antigen in paraffin section. Detection of cytoplasmic inclusion bodies. *Jpn J. Exp. Med.*, **44**, 25.

SUMITHRAN, E. & MACSWEEN, R. N. M. (1979) An appraisal of the relationship between primary hepatocellular carcinoma and hepatitis B virus. *Histopathology*, **3**, 447.

WORLD HEALTH ORGANIZATION (1955 & 1969) *Manual of the International Classification of Diseases, Injuries and Causes of Death.* 7th & 8th Revisions. Geneva: WHO.

WORLD HEALTH ORGANIZATION (1973) Viral hepatitis. Report of a WHO Scientific Group. *WHO Techn. Rep. Series*, No. 512.

WU, P. C. (1978) Detection of hepatitis B surface antigen in liver biopsies from 655 Chinese patients in Hong Kong. *Asian J. Infect. Dis.*, **2**, 223.

WU, P. C. & LAM, K. C. (1979) Cytoplasmic hepatitis B surface antigen and the ground-glass appearance in hepatocellular carcinoma. *Am. J. Clin. Pathol.*, **71**, 229.